# Draft genome sequencing of *Pediococcus acidilactici* strains isolated from cow’s milk: unlocking insights into functional traits and applications

**DOI:** 10.1128/mra.00252-24

**Published:** 2024-07-18

**Authors:** Naim Siddique, Md. Morshedur Rahman, Salma Akter, Mehedi Mahmudul Hasan, Ziban Chandra Das, M. Nazmul Hoque

**Affiliations:** 1 Molecular Biology and Bioinformatics Laboratory, Department of Gynaecology, Obstetrics and Reproductive Health, Bangabandhu Sheikh Mujibur Rahman Agricultural University (BSMRAU), Gazipur, Bangladesh; 2 Department of Microbiology, Jahangirnagar University, Dhaka, Bangladesh; 3 Department of Fisheries and Marine Science, Noakhali Science and Technology University, Noakhali, Bangladesh; SUNY College of Environmental Science and Forestry, Syracuse, New York, USA

**Keywords:** draft genome, probiotics, *Pediococcus acidilactici*, cow's milk

## Abstract

*Pediococcus acidilactici* is a potential probiotic bacteria isolated from diverse sources. However, strains isolated from milk, especially from raw milk of healthy cows, have not been thoroughly studied. Here, we report the draft genome sequence of *P. acidilactici* strains MBBL5 and MBBL7, isolated from milk samples of healthy cows.

## ANNOUNCEMENT

*Pediococcus acidilactici*, isolated from various environments, is gaining attention as a probiotic for its resilience to extreme conditions and antimicrobial properties (1 – 3). Different strains of *P. acidilactici* produce pediocins (class II bacteriocin), which can inhibit various pathogens (4, 5). Several studies have explored their applications in biopreservation, as well as in human and veterinary medicine (5 – 7).

Milk samples were collected from lactating cows at the Bangabandhu Sheikh Mujibur Rahman Agricultural University dairy farm situated in Gazipur (24.09° N, 90.41° E), Bangladesh. California mastitis test was used to confirm the absence of mastitis in these cows (8). Following overnight incubation in deMan, Rogosa and Sharpe (MRS) broth (HiMedia, India) at 37°C, enriched milk samples were plated on MRS agar (Merck, Germany) and incubated for 48 hours at 37°C. Phenotypic identification involved assessing colony morphology (small, creamy, and smooth colonies), Gram staining (Gram-positive, spherical cells), and biochemical tests (catalase and oxidase negative) (9). Further confirmation of *P. acidilactici* isolates was performed through VITEK-2 system v.9.01 (10). Isolates MBBL5 and MBBL7 of *P. acidilactici* were subcultured in nutrient broth at 37°C overnight, followed by DNA extraction using QIAamp DNA Mini Kit (QIAGEN, Germany). The purity and concentration of the extracted DNA were assessed using NanoDrop 2000 (Thermo Fisher Scientific, USA) (11). Libraries from 1 ng of DNA were generated using Nextera DNA Flex kit (Illumina, San Diego, USA), followed by whole-genome sequencing on the Illumina MiSeq sequencer using a 2 × 250 bp protocol. Trimmomatic v.0.39 (12) was utilized to remove Illumina adapters, known Illumina artifacts, and phiX reads from the raw reads (MBBL5 = 4,083,696; MBBL7 = 4,842,056) of both isolates. Following quality assessment with FastQC v0.11.7 (13), reads exceeding a Phred score of 20 (14) were assembled using SPAdes v.3.16 (15). Genome annotation was performed using the National Center for Biotechnology Information Prokaryotic Genome Annotation Pipeline v.6.6 (16), along with the *Pediococcus* CheckM v.1.2.2 marker set (17). The detection of antimicrobial resistance genes (ARGs), virulence genes, bacteriocin genes, prophages, and plasmids and the prediction of secondary metabolites in both genomes were performed using CARD v.3.2.4 (18), VirulenceFinder v.2.0 (19), BAGEL4 (20), PHASTER (21), PlasmidFinder (22), and AntiSMASH v.7.0 (23) databases, respectively. Default parameters were used for all software unless otherwise specified.

The draft assembly sizes for *P. acidilactici* strains MBBL5 and MBBL7 were 2,028,504 and 2,021,980 bp, comprising 57 and 51 contigs, and 1,902 and 1,900 predicted genes, respectively. Additional sequencing, assembly, and annotation statistics for both genomes are detailed in Table 1. Both genomes were found to harbor two ARGs (*van*T and *qac*G) and one prophage region. Notably, MBBL5 contained two plasmids (LBPp6 and pR18), whereas MBBL7 carried a single plasmid (LBPp6). However, no bacteriocin gene clusters and secondary metabolite regions were detected in these genomes. Therefore, *P. acidilactici* strains from healthy cow’s milk, potentially lacking specific resistance genes, may serve as promising bioactive probiotics to reduce disease and enhance dairy product development.

**TABLE 1:** Genomic features of the *P. acidilactici* stains MBBL5 and MBBL7

Features	*P. acidilactici* strains	
	MBBL5	MBBL7
Genome size (bp)	2,028,504	2,021,980
Genome coverage (*x*)	108	134.3
GC content (%)	42.03	42.04
No. of contigs	57	51
*N*_50_ (bp)	137,427	167,005
Protein coding genes	1,902	1,900
No. of plasmids	2	1
Genome completeness (%)	96.09	96.09
Genome accession numbers	JAZIFQ000000000	JAZIFS000000000
Sequence Read Archive accession numbers	SRR27558661	SRR27558659

## Data Availability

The draft genomes of Pediococcus acidilactici strains MBBL5 and MBBL7 are available at the National Center for Biotechnology Information/GenBank under BioProject (accession number PRJNA1065156) and Sequence Read Archive (accession numbers SRR27558661 and SRR27558659). Individual accession numbers are listed in Table 1. The versions described in this paper are JAZIFQ000000000.1 and JAZIFS000000000.1.
